# Recent Advances in 3D Printing of Polymers for Application in Prosthodontics

**DOI:** 10.3390/polym15234525

**Published:** 2023-11-24

**Authors:** Mariya Dimitrova, Angelina Vlahova, Yavor Kalachev, Stefan Zlatev, Rada Kazakova, Saverio Capodiferro

**Affiliations:** 1 Department of Prosthetic Dentistry, Faculty of Dental Medicine, Medical University of Plovdiv, 4000 Plovdiv, Bulgaria; angelina.vlahova@mu-plovdiv.bg (A.V.); yavor.kalachev@mu-plovdiv.bg (Y.K.); stefan.zlatev@mu-plovdiv.bg (S.Z.); rada.kazakova@mu-plovdiv.bg (R.K.); 2 CAD/CAM Center of Dental Medicine, Research Institute, Medical University of Plovdiv, 4000 Plovdiv, Bulgaria; 3 Department of Interdisciplinary Medicine, Aldo Moro, University of Bari, 70100 Bari, Italy; saverio.capodiferro@uniba.it

**Keywords:** 3D printing, prosthodontics, additive manufacturing, polymers, digital, prosthetic restorations

## Abstract

Contemporary mass media frequently depict 3D printing as a technology with widespread utilization in the creation of dental prosthetics. This paper endeavors to provide an evidence-based assessment of the current scope of 3D printing’s integration within dental laboratories and practices. Its primary objective is to offer a systematic evaluation of the existing applications of 3D-printing technology within the realm of dental prosthetic restorations. Furthermore, this article delves into potential prospects, while also critically examining the sustained relevance of conventional dental laboratory services and manufacturing procedures. The central focus of this article is to expound upon the extent to which 3D printing is presently harnessed for crafting dental prosthetic appliances. By presenting verifiable data and factual insights, this article aspires to elucidate the actual implementation of 3D printing in prosthetic dentistry and its seamless integration into dental practices. The aim of this narrative review is twofold: firstly, to provide an informed and unbiased evaluation of the role that 3D printing currently plays within dental laboratories and practices; and secondly, to instigate contemplation on the transformative potential of this technology, both in terms of its contemporary impact and its future implications, while maintaining a balanced consideration of traditional dental approaches.

## 1. Introduction

The advent of industrial-scale additive manufacturing, more commonly known as 3D printing, saw its initial appearance on the market during the early 1980s [1]. Visionaries in the realm of 3D printing include notable figures like Charles W. Hull (the visionary behind 3D Systems), S. Scott Crump (the mind behind Stratasys), and the pioneering duo of Hans J. Langer and Hans Steinbichler (the founders of EOS). It was Charles W. Hull who obtained the patent for the first 3D printer in 1986 [2]. In their nascent phase, these 3D printers primarily found their utility in the domain of rapid prototyping [3,4].

Nevertheless, the evolution of this technology surged forward with remarkable speed in the subsequent years [5]. Notably, the expiry of the patent for the fused deposition modeling (FDM) process in 2009 propelled 3D printers into the realm of consumers, catalyzing a significant penetration into this sector [6,7]. This transformative momentum extended its influence into the realm of dentistry as well [8]. The dimensions of printing units shrank while costs diminished, resulting in an altered landscape of applicability [9,10]. Simultaneously, the gamut of printable materials broadened to encompass a diverse array, ranging from plastics and metals to ceramics and even biological tissues [11]. These rapid prototyping methods can be classified based on the specific types of materials employed, such as plastics, metals, or powders [12,13].

Additive manufacturing (AM) procedures involve the step-by-step creation of items according to three-dimensional models [3]. The phrase commonly employed interchangeably with all additive techniques is 3D printing. As per the EN ISO/ASTM 52900:2021 standard for terminology, an AM process is defined as the “procedure of combining materials to construct objects using 3D model data, typically layer by layer, in contrast to subtractive manufacturing techniques” [9].

The speed of progress in the evolution of digital dental manufacturing has become truly remarkable [14]. Subtractive methods have achieved remarkable levels of both efficiency and precision in achieving accurate fits, while additive techniques such as 3D printing are gaining prominence at an escalating rate [15]. The merging of various manufacturing approaches, such as the pairing of laser sintering with CNC machining, or the integration of digital design and 3D printing alongside traditional analog pressing, vividly showcases the immense possibilities that lie ahead [16,17].

Societal shifts are exerting transformative influences on the field of dental technology, much like they do in other sectors [18]. One of these profound changes pertains to a scarcity of a proficient workforce. Notably, there is an ongoing decline in the number of individuals pursuing training in dental technology [19], even as the demand for dental prostheses continues to surge due to shifting demographics [8]. Furthermore, patients are increasingly constrained by time limitations prompted by heightened expectations in their professional spheres, curtailing their capacity to undergo dental procedures [20].

In the face of these challenges, the digital revolution emerges as a potential solution, as digital procedures often stand out for their efficiency [21]. Within the dental laboratory, the integration of digital processes offers advantages like heightened precision and reproducibility, alongside enhanced material characteristics and user convenience [22]. This technological transition holds the potential to address these challenges by harnessing the inherent efficiencies of digital methodologies [23,24].

The intriguing fusion of a digitalized work environment and a hands-on artisanal craft renders dental technology a compelling choice for young individuals seeking a diverse and multifaceted professional journey [25]. Numerous dental laboratories have adeptly embraced the delicate equilibrium between preserving age-old craftsmanship and embracing the digital realm, finding harmony between tradition and disruption, and reconciling established values with the need for necessary changes [26,27]. Within this unfolding narrative, the role of 3D printing as a digital manufacturing process takes center stage, illuminating a pivotal facet of this evolution [28]. To put it in simplified terms, the workflow involves a dental technician generating a digital dataset on a computer and creating a three-dimensional entity through computer-aided design (CAD) [29]. This digital blueprint is then transmitted to a 3D printer, where it metamorphoses into a tangible object [30].

An inherent advantage of additive manufacturing techniques lies in their capacity to visualize and actualize three-dimensional concepts on a screen, enabling the realization of a virtually boundless array of forms and intricacies [31]. An intriguing facet often overlooked is that the mechanical and aesthetic attributes of the fabricated item can be subtly tailored during the 3D construction process [32]. This distinct capability is absent in subtractive manufacturing, wherein the material characteristics are predetermined by the supplier of the prefabricated material [33,34]. The confluence of customization opportunities and the expeditious availability of digitally conceived items, often at reduced costs, positions additive manufacturing as a pivotal cornerstone within the realm of digital dentistry [35,36].

Polymeric materials play a significant role in the realm of dentistry, offering a wide array of applications stemming from their distinct surface qualities, mechanical and biological attributes, simplified processing, and cost-effectiveness [4]. Among the frequently employed polymers in dental applications are polymethyl methacrylate (PMMA), polyurethane (PU), polyethylene (PE), polycarbonate (PC), polyetheretherketone (PEEK), polyethylene glycol (PEG), polydimethylsiloxane (PDMS), polylactic acid (PLA), poly(e-caprolactone) (PCL), acrylonitrile butadiene styrene (ABS), and polypropylene (PP) [2].

While their mechanical traits are linked to the inherent properties of the bulk material, their interaction with oral tissues heavily relies on surface characteristics. This justifies the utilization of polymer coatings to enhance their biocompatibility [7]. The applications of these polymers span nearly all sectors of dentistry, encompassing direct restorative procedures, prosthodontics, orthodontics, and even implantology. Notably, synthetic PEEK has emerged as a potential implant material [15]. Leveraging 3D printing, intricately detailed custom facial prostheses made from polymers can be readily produced [26]. Furthermore, polymers have been instrumental in crafting scaffolds that contribute to bone structure regeneration, along with the development of tissues resembling dentin and pulp. They also find utility in producing membranes for guided tissue regeneration and as carriers for drug delivery in the treatment of various oral and periodontal conditions [21].

**Figure 1:**
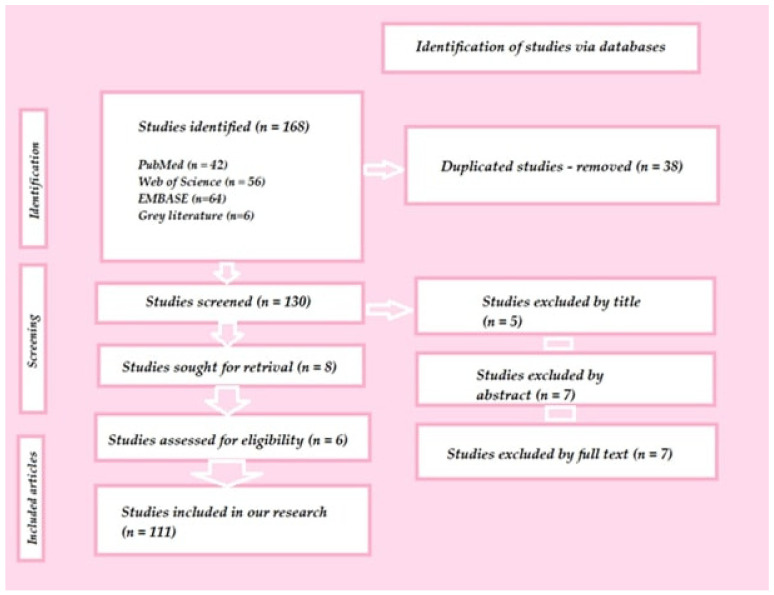
Design of the conducted study.

The objective of this narrative review is to provide an overview of the current advanced status of additive manufacturing within prosthodontics, with a specific focus on polymeric materials, and to outline potential future perspectives for this modern technology. To achieve this, a comprehensive search was conducted across prominent databases including PubMed, Web of Science, and EMBASE (Figure 1).

The scope of the search encompassed articles that documented the utilization of 3D-printed polymers in prosthetic dentistry. This was accomplished by employing a combination of diverse keywords, such as “dentistry” OR “digital dentistry”, AND “polymers”, AND “3D printing” OR “rapid prototyping” OR “additive manufacturing” OR “digital prosthodontics”. The inclusion criteria ensured that only full-text articles written in English were considered for analysis. In addition to the electronic search, a supplementary manual exploration of relevant citations and references was carried out to further augment the comprehensiveness of the review.

## 2. General Techniques for 3D Printing in Prosthetic Dentistry

**Figure 2:**
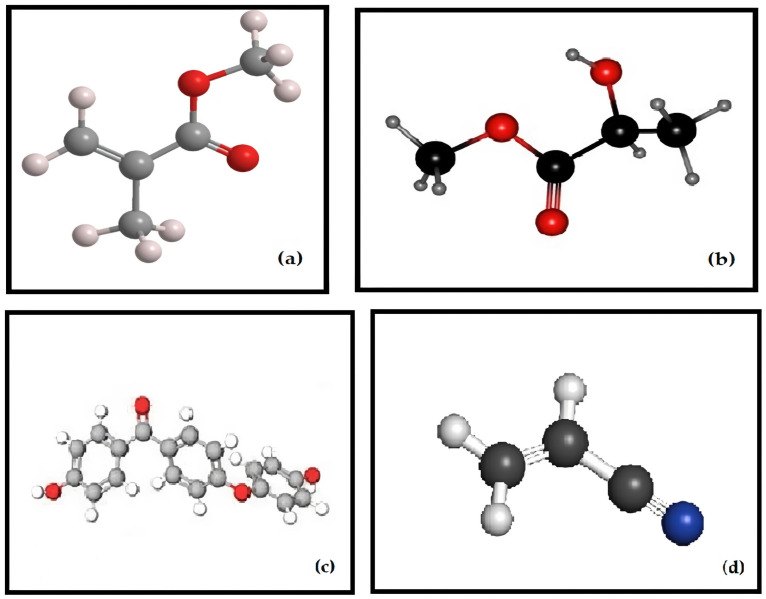
Molecular structures of the polymers for 3D Printing in Prosthodontics—(**a**) PMMA; (**b**) PLA; (**c**) PEEK; (**d**) ABS.

**Figure 3:**
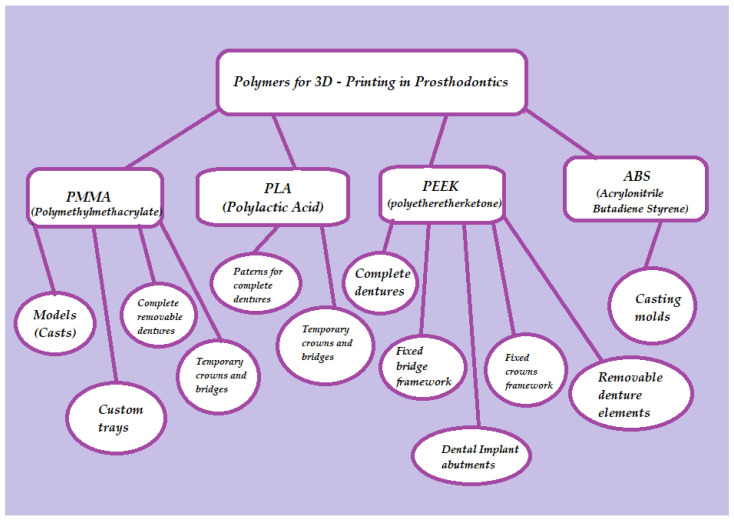
Three-dimensional-printed polymers used in prosthetic dentistry and their application.

Among the various dental specialties, prosthodontics appears to have reaped substantial benefits from the advancements in 3D-printing technology. This is particularly evident in both fixed and removable denture fabrication, which can now be seamlessly executed through a fully digital workflow, resulting in precise and well-fitting prostheses. Polymers have proven to be well-suited for additive manufacturing processes involved in creating temporary crowns, denture bases, and artificial teeth. There have even been efforts to explore the 3D printing of polymeric permanent crowns and bridges. The range of applications of 3D-printed polymers in prosthodontics extends to crafting custom trays, patterns, try-ins, dental bite registrations, and various types of models. Several frequently employed polymers in the field of prosthetic dentistry include polymethylmethacrylate (PMMA), polylactic acid (PLA), polyetheretherketone (PEEK), and acrylonitrile butadiene styrene (ABS). Their molecular structures and general applications in the prosthodontic field are presented in Figure 2 and Figure 3.

**Figure 4:**
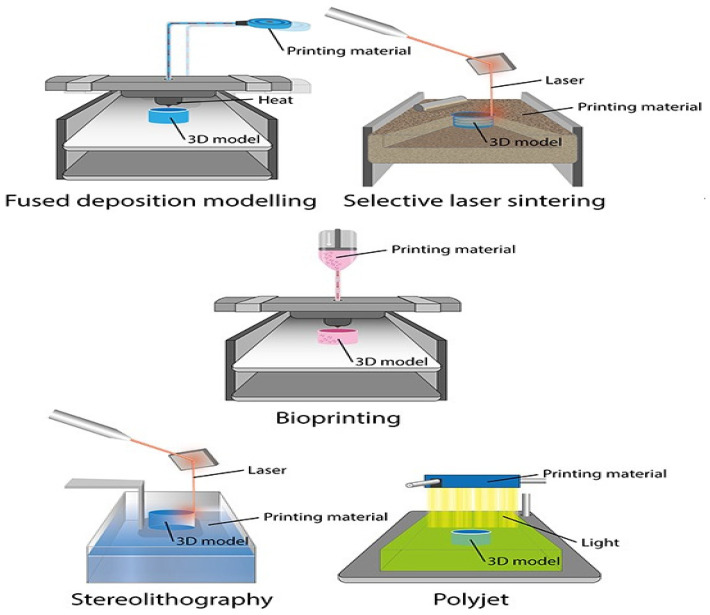
Application of 3D printers in dentistry.

**Figure 5:**
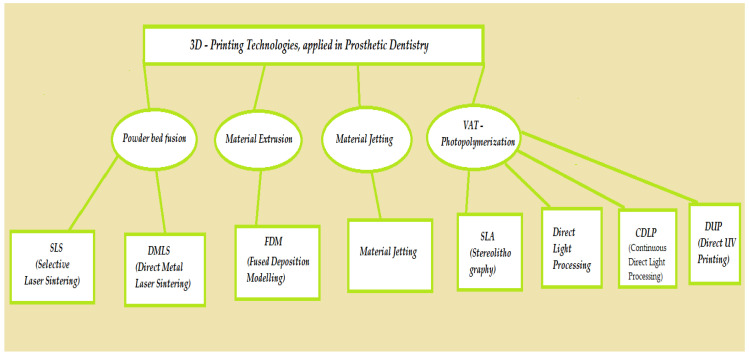
Three-dimensional-printing technologies, applied in prosthetic dentistry.

Multiple 3D printing methods are available for the additive fabrication of prosthetic restorations (Figure 3, Figure 4 and Figure 5). 

When these methods are juxtaposed, they reveal distinct attributes, encompassing speed, precision, scale, and procedural stability, contingent upon the underlying technology employed [37,38]. At present, within the domain of prosthetic dentistry, stereolithographic techniques emerge as the prevailing choice [39]. These encompass conventional stereolithography, laser-based solidification (stereolithography, SLA), and mask exposure methods (digital light processing, DLP). In both these methodologies, the object is solidified within a reservoir of photopolymer through the influence of light [40,41].

Starting in 2018, an array of 3D printers has entered the market that utilizes economical liquid crystal displays (LCD) [42]. This technology is termed direct ultraviolet printing (DUP) and exploits the LCD screens for precise pixel-wise exposure of the construction platform [43]. The typical choice for illumination is UV LEDs within a wavelength range of 395 to 405 nm. Moreover, direct 3D printing processes, also known as material jetting (MJT), have found application within dental contexts. Notably, there is a noteworthy technique known as multi-material 3D printing by Stratasys, which enables the simultaneous processing of diverse colors and materials with varying properties in a single build [5,44].

However, within the dental field, material extrusion (MEX) processes, including techniques such as fused-filament fabrication (FFF) or fused deposition modeling (FDM), currently face drawbacks as indicated by recent findings [45]. This is attributed to the extended printing times and challenges in attaining higher resolutions associated with these processes [46]. Among the diverse technologies mentioned within the realm of plastics, it is apparent that SLA, DLP, and MJT stand out as particularly compelling from both a technical and economic perspective [47,48]. Table 1 presents the most commonly used polymer materials in prosthodontics, organized according to their processing and their properties (Table 1).

The pioneering 3D printing systems to enter the market were stereolithographic setups that employ laser beams to solidify liquid substances [49]. Charles Hull, as early as the 1980s, filed a patent application for the initial stereolithography printer [50]. In their initial iterations, these devices were notably extensive and came with a high price tag [51]. In contrast, the latest wave of stereolithographic printers has become significantly more cost-effective. For the past five years, Formlabs (Sommerville, MA, USA) has provided a 3D printer tailored for dental applications [52]. This budget-friendly system serves as an excellent point of entry into 3D-printing technology, although it should be noted that the printing process might take longer compared to DLP printers [53,54].

In conjunction with stereolithography, digital light processing (DLP) stands out as one of the most widely adopted additive manufacturing methods within the current dental domain [55]. The configuration of a DLP printer closely resembles that of an SLA printer, with the primary distinction being the source of illumination utilized [56,57]. Unlike the SLA printer that employs a laser beam for curing the photopolymer, DLP printers employ projection technology developed by Texas Instruments [58]. In this method, short-wavelength light (currently operating at wavelengths of 380 nm and 405 nm) is directed through a central digital micromirror device (DMD), which constitutes the core of DLP technology [59,60]. This setup employs micro mirrors with an approximate edge length of 16 µm that can be individually tilted under the influence of electrostatic forces, enabling the light to be optically directed onto the build platform [61]. This platform is situated within a translucent reservoir of photopolymer (also known as a photopolymer bath), or onto an absorptive surface [62,63]. The light, channeled through the DMD, projects the exposure mask onto the build platform through an optical lens. This prompts the photopolymer to solidify in the exposed regions [64]. After each mask projection, the build platform ascends along the z-axis, allowing fresh material to flow into the area beneath the object, thereby preparing it for exposure with the next mask [65,66]. In the realm of DLP technology, the time required for fabrication is predominantly determined by the object’s dimension along the z-axis, rendering it relatively independent of the object’s overall complexity or geometry [67].

In DLP (digital light processing) printers, the process involves using tiny mirrors to create individual image points or pixels [68]. However, the number of these micromirrors on a DMD (digital micromirror device) is limited. This limitation becomes evident when enlarging the build platform, which consequently increases the lengths of the edges along the x and y axes [69,70]. This expansion results in reduced precision. Despite this challenge, there are three methods currently employed to achieve larger build platforms. Inexpensive DLP printers utilize DMD chips with lower resolutions, leading to a smaller physical size. By using high-resolution DMD chips (e.g., HD 1920 × 1080 pixels), greater object accuracy can be attained without increasing the printer’s footprint [71]. Even more impressive, the use of 4K DMD chips (3840 × 2160 pixels) enables the combination of high resolutions with a sizable build area, as seen in the Rapid Shape D70+ printer. However, the cost of 4K DMD chips remains a significant obstacle.

The approach of running two DLP projectors with HD resolution in parallel results in a seam or “joint” on the build platform due to the utilization of two light sources. This joint prevents the printing of objects that span the projection field. For instance, the Rapid Shape D40 II printer employs this method [72]. To enhance the capabilities of DLP printers, innovations are applied in detaching objects from the material vat during the build process (W2P Engineering, Vienna, Austria). This detachment occurs after each cycle of exposure when the build platform is raised along the z-axis [73]. Four distinct techniques are employed to achieve these fixed intervals. The build platform follows a predefined path within a specific timeframe after each exposure cycle. This approach maintains a consistent path-to-time ratio throughout the build process, even if fewer support structures could allow for earlier detachment. While straightforward, this method does not alter the overall duration of the building processes [74].

Force sensors are utilized to measure the detachment force required (Rapid Shape, Heimsheim, Germany). Smart control technology calculates an optimal path-to-time ratio, thus accelerating the building process. An important benefit is the precise and controlled separation process. The patented Force Feedback technology is exemplified in printers like the Rapid Shape D30 [75].

Vat Deflection Feedback System (VDFS; W2P, Vienna, Austria) employs an additional sensor to expedite the building process. By allowing the deformation of the material tray (FlexVat), the detachment force is minimized. This leads to heightened printing speed and quality [76].

Carbon3D (CDLP; Carbon3D, Redwood City, CA, USA) introduced the patented continuous liquid interface production (CLIP) technology, categorized as a CDLP process. Unlike the step-by-step object buildup in conventional DLP printers, CLIP employs a continuous build process that does not necessitate the usual detachment steps. This is achievable due to an oxygen-rich zone (“dead zone”) immediately above the build platform, where the photopolymer does not cure. Oxygen is introduced into this zone through an oxygen-permeable window [77]. As adhesion between the object and the build platform is absent, a continuous build process becomes feasible. The outcome is rapid build speeds, precise object formation, and uninterrupted object geometries along the z-axis. Examples of dental applications encompass digitally manufactured Lucitone Digital Print denture bases from Dentsply Sirona (York, PA, USA), as well as bite splints produced using Carbon3D printers like KeyPrint and KeySplint Soft Clear [78].

DLP projectors that move underneath the material vat can expose a larger area for printing. A key advantage of this approach is the absence of a joint line on the printed object. As a result, the entire build platform can be used at full resolution, leading to higher precision, increased printing accuracy, and optimal utilization of the printer’s capacity [79].

MovingLight (Prodways Group, Paris, France) is a technology based on the DLP process but stands out due to its dynamic projector movement. Unlike its competitors, the projector is not fixed in a single position within the printer; instead, it moves across the entire working area in multiple steps [80]. This innovative movement results in high resolutions (42 µm) and remarkable accuracy, even with a substantial build platform. Examples of printers utilizing this technology include Prodways’ ProMaker LD10 Dental Plus, LD10 Dental Models, LD20 Dental Plus, and LD20 Dental Models. The latter two models incorporate two movable projector heads, reducing printing times by an additional 40%. For instance, these printers can produce 55 dental arches in approximately 1 h [81,82].

Another approach is the material jetting technique, in which the material is deposited directly onto the build platform using a print head, resembling the process of 2D printing. Following this deposition, the material undergoes curing during an intermediate exposure step, gradually constructing the object layer by layer. The most prominent example of this approach is the Polyjet method, developed by Stratasys in Eden Prairie, MN, USA [83]. This method is characterized by its rapid building process and remarkable precision. Notably, it supports multi-material 3D printing, allowing for the creation of objects using up to five distinct materials in a palette of over 500,000 colors. Stratasys offers products like the J720 Dental and J750 Digital Anatomy printers, both capable of operating in multi-material and multicolor modes [84].

At present, polymers stand as the predominant choice for 3D printing within the dental field. They are employed to create both permanent and removable dentures, along with a diverse array of dental devices, dental implants, and tissue formations.

## 3. Applications of 3D-Printed Polymers in Fixed Prosthodontics

### 3.1. Fabricating of 3D Models Using Intraoral Scan

**Figure 6:**
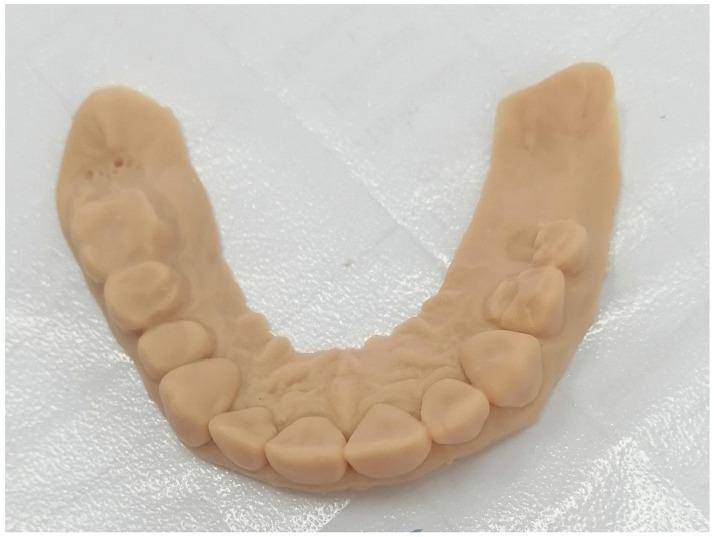
Three-dimensional–printed model of an upper jaw (origin of the Figure: author’s own clinical case, no copyright issue).

Thanks to the impressive efficiency and precision of DLP printers, they excel in fabricating master models and segmented models from intraoral scan data. This capability finds significant utility in various applications, with a notable one being in oral implantology [85]. In this context, producing models for oral implantology using DLP printers holds promise. A crucial aspect of this process involves accurately placing laboratory analogs within the printed model, as this directly impacts the fit of restorations, both proximally and occlusally (Figure 6) [86].

### 3.2. Fabricating of Temporary Crowns and Fixed Partial Dentures

Temporary crowns and bridges play a crucial role in the process of treating fixed partial dentures (FPDs). Their primary purposes include safeguarding the pulp, ensuring alignment with the bite, preserving tooth placement, preventing fractures, and enduring functional pressures [87]. On the aesthetic front, they must exhibit a lasting color and the appropriate level of translucency. Provisional crowns need to exhibit exceptional functionality along with strong aesthetic and preventive qualities. Moreover, they serve as prototypes for future fixed partial dentures (FPDs) [88].

**Figure 7:**
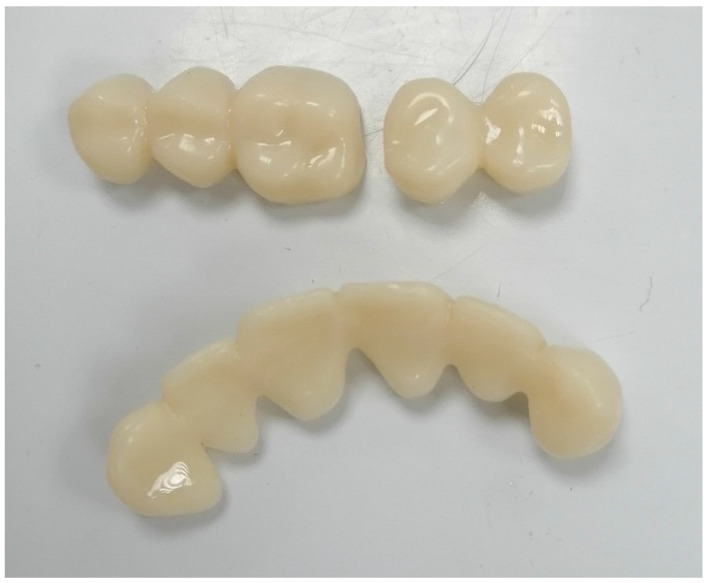
Three-dimensional-printed provisional bridges (origin of the Figure: author’s own clinical case, no copyright issue), (Temporary CB Resin, Formlabs Dental, Somerville, MA, USA).

Currently, a diverse range of materials and technologies are employed in the fabrication of temporary FPDs to ensure simple production, pleasing aesthetics, and relatively elevated levels of hardness and strength [89]. The materials used for crafting provisional crowns and bridges can be divided into two primary categories: (1) methacrylate polymers and (2) composites. Polymethyl methacrylate (PMMA), polymethyl methacrylate, polyvinyl ethyl methacrylate, bisphenol A glycidyl methacrylate, urethane dimethacrylate, and others are the key materials in use [90]. The methods employed for producing temporary crowns and bridges encompass traditional techniques, involving heat-cured PMMA, as well as the relatively modern CAD-CAM milling of PMMA blanks [91]. Heat-cured acrylic polymers exhibit greater strength and resistance to wear compared to self-cured alternatives. Additionally, they display consistent color and are easily treatable on the surface. Temporary structures created from these materials can effectively fulfill their function over extended periods (Figure 7).

Utilizing CAD-CAM systems to mill provisional prostheses enables the utilization of high-density polymers, ensuring relatively robust mechanical characteristics and biocompatibility [92]. This results in the rapid creation of temporary restorations with notable precision concerning anatomical form, adherence to the teeth, and occlusal interactions [93]. These clear advantages offered by CAD-CAM systems have led to their extensive adoption within dental clinics and laboratories in recent times. Incorporating 3D-printing technology into the CAM module, along with the partial or complete digitization of processes right from the initial impression, can significantly reduce production time and deliver temporary prosthetic restorations of requisite accuracy and quality [94].

In the research by Digholkar S. et al. [95], an investigation was conducted into the microhardness and flexural strength of substances used in the production of conventional temporary fixed partial dentures (FPDs). These materials were derived from heat-cured polymer, PMMA produced through CAD-CAM milling, and a microhybrid photo-cured composite fabricated using 3D-printing technology. Their findings revealed that among the three materials, CAD-CAM-milled polymer exhibited the highest bending strength at 104.20 Mpa [96]. Conversely, in terms of microhardness, the 3D-printed composite displayed the greatest hardness at 32.77 HKN, attributed to the presence of filler. Tahayeri A. and collaborators generated samples using the commercially available NextDent C & B Vertex Dental polymer intended for temporary crowns and bridges [97]. They utilized a cost-effective Form 1+ printer from FormLabs, based on laser stereolithography (SLA) principles. Despite the limited precision of the printing system, they established that SLA-fabricated materials for temporary restorations possessed the essential mechanical characteristics for intraoral application [98].

Mai HN et al. [99] indicated that crowns produced through CAD-CAM systems (both milling and polymer-jet 3D printing) exhibited greater fitting accuracy compared to those created using matrices. This heightened accuracy, particularly in the occlusal region, was attributed to the significant enhancement in precision achieved through polymer-jet 3D printing. Additionally, Kim DY et al. [100] pointed out that the precision of dental crown fittings is impacted by the quantity of samples manufactured through microstereolithography. The most precise details are obtained when three pieces are printed on a single platform.

### 3.3. Fabricating Permanent Crowns, Inlays, Onlays, and Veneers

**Figure 8:**
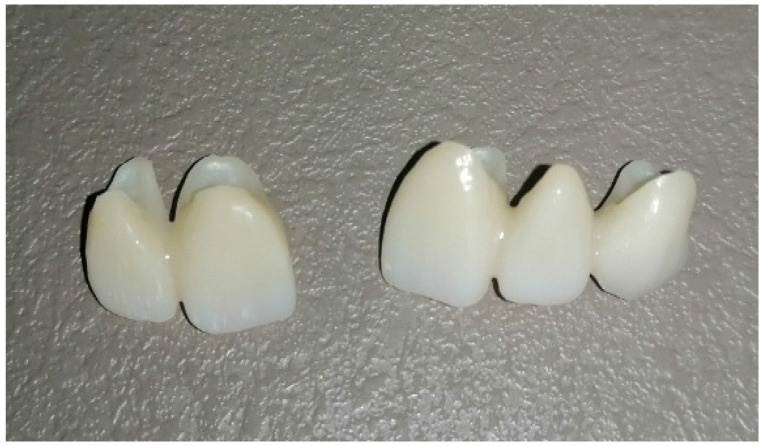
Permanent resin block crowns (origin of the Figure: author’s own clinical case, no copyright issue), (Permanent Crown Resin, Formlabs Dental, USA).

A ceramic-infused resin (Permanent Crown Resin, Formlabs Dental, USA) with a natural tooth color has been designed for 3D printing of durable single crowns, inlays, onlays, and veneers (Figure 8) [101]. The Permanent Crown Resin creates resilient and accurate long-term dental restorations, offered in four VITA Classical shades. The minimal water absorption and smooth surface guarantee that restorations crafted from this resin resist aging, discoloration, and plaque buildup [102].

### 3.4. Fabricating of Permanent Post and Core Restorations

**Figure 9:**
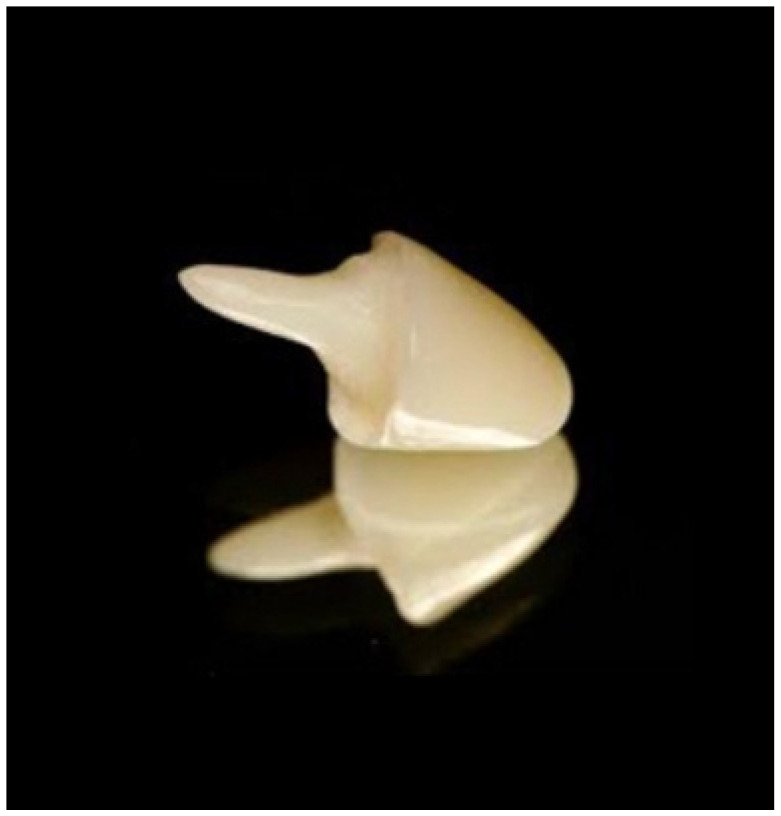
Post and core restoration, fabricated by PEEK (origin of the Figure: author’s own clinical case, no copyright issue).

When dealing with significant damage to the crown part of a tooth, necessitating added retention, insertion of a post into the root becomes essential to secure both the core and the restoration (Figure 9) [5,103]. The decision between utilizing a post that is tailor-made or one that is pre-manufactured depends on several considerations, including the shape of the canal, the amount of tooth structure remaining, and the chosen method of restoration [27].

Custom-designed posts are predominantly crafted from materials such as metal, zirconia, and composite materials reinforced with fibers.

As per the existing literature, the ideal choice of material for crafting post and core components should possess an elastic modulus that aligns with the natural flexural dynamics of the root [4,6,10]. Employing materials with biomechanical properties resembling dentin could also serve to mitigate the risks of root fractures or detachment [21]. One such category of materials demonstrating these attributes is advanced polymers like polyetheretherketone (PEEK) [104].

Past research has indicated that polyetheretherketone (PEEK) stands out as a promising biocompatible substance characterized by effective shock absorption, mechanical robustness, and notable resistance to both heat and chemicals [17]. Polyetheretherketone (PEEK) stands as a high-performance semi-crystalline polymer renowned for its impressive biocompatibility and strong processability [2]. PEEK exhibits substantial promise as a material for oral prosthetics due to its lightweight nature and relatively lower modulus (3–4 Gpa), rendering it a viable substitute for traditional Co-Cr alloy (230 Gpa) and Ti (104 Gpa). Limited studies have explored the application of PEEK in post and core assemblies [15,22]. In theory, utilizing PEEK and similar materials for post and core structures could potentially reduce the occurrence of root fractures [105]. To substantiate this hypothesis, additional laboratory and clinical investigations are imperative.

Beyond the appropriate biomaterial, the integration of a digital workflow through CAD/CAM methodologies also assumes significance in refining the creation of custom-designed post and core elements [9,13]. This approach permits the digital planning of anatomical structures, followed by milling or 3D printing [4]. The utilization of CAD/CAM technology not only streamlines the post and core fabrication process, saving time, but also enhances reliability [11]. Moreover, CAD/CAM-enabled post and core systems enable meticulous control over design aspects and the thickness of the cement layer [4].

Three-dimensional printing demonstrates a remarkable capability in handling thermoplastics, including PEEK, showcasing heightened production efficiency and minimal material wastage in contrast to traditional subtractive techniques [106]. Recent investigations have introduced the concept of dual-nozzle printing technology for processing diverse materials, thereby potentially supporting the realization of dual-color 3D printing for oral prosthetic materials that emulate tooth and gingiva shades [13]. Although 3D-printed PEEK has been successfully employed in various cases involving implants, artificial ribs, and frameworks for removable prostheses, its use remains relatively limited, and the application of dual-color PEEK printing in dental contexts remains unexplored [12,16]. A constraint of 3D-printed PEEK is its inherent brown color, negatively impacting aesthetics and hindering broader adoption [19].

According to the study of Chen et al. [107], this limitation can be overcome by fabricating a speech aid prosthesis framework using titanium dioxide (TiO2)/PEEK composite, achieving augmented mechanical strength and enhanced aesthetics. Removable dental prostheses necessitate both tooth-colored and gingival-colored components to mimic the textures of hard and soft oral tissues [108]. However, a monochromatic PEEK material falls short of capturing both tooth and gingiva colors simultaneously [14]. Apart from compromised aesthetics, PEEK frameworks require additional post-printing procedures like casting and molding to create the final prosthesis. Conventional manufacturing processes are not only time-intensive but also technologically demanding. Moreover, the interface between distinct parts cannot be eliminated as effectively as in one-piece printing with a dual-nozzle setup.

### 3.5. Fabricating of Drilling Stents for Guided Implantology

Recent software advancements have facilitated the integration of volumetric data sets derived from radiology (DICOM) with surface data sets (STL) obtained from laboratory or intraoral scanners. This integration enables the enhancement of implant placement, considering anatomical, surgical, and prosthetic considerations. Subsequently, these planned positions are executed using a surgical template that is placed within the patient’s oral cavity [8]. DLP printing technology stands out in this context due to its ability to rapidly produce these templates with cost-effectiveness. Unlike subtractive techniques, DLP printing allows for the creation of intricate three-dimensional geometries without limitations on design possibilities [13].

## 4. Applications of 3D-Printed Polymers in Removable Prosthodontics

### 4.1. Manufacturing of Custom Trays

DLP printing technology presents a compelling option for producing personalized impression trays, primarily due to its swift processing. CAD software (DentalCAD 3.1 Rijeka, Exocad GmbH, Darmstadt, Germany) solutions available in the market enable the design of custom impression trays with optimal fit parameters in a streamlined manner, leading to significant time savings [11]. This is especially true when virtual undercut blocking and precise dimensional adjustments are factored in. Ensuring the impression’s removal without irreversible deformation is critical [63]. Despite the technical benefits, it is worth noting that materials currently designated for crafting functional impression trays come at a high cost [86]. As a result, their practicality is primarily seen in the realm of implant impression trays. To make the most of this technology, it is advisable to integrate it with digital implant planning. In this scenario, digital models are already available, and the planned implant positions can serve as a foundation for constructing the trays.

### 4.2. Manufacturing of Denture Bases for Removable Dentures

Removable dentures are possible to be created using stereolithography (SLA) with 3D printers, which exhibit enhanced precision when the printing direction is inclined at a 45° angle [84]. When it comes to complete prosthetics, the concept of the impression technique holds appeal, but there is still a need for advancements in both materials and methodologies [6,85]. The complete digital production of removable partial dentures (RPDs) is presently limited to cases falling within the Kennedy III/IV classifications. For partially edentulous scenarios classified as Kennedy class I/II, the digital impression technique struggles to accurately capture the base edges and the displacement of the mucosa when the prosthesis applies pressure [86].

**Figure 10:**
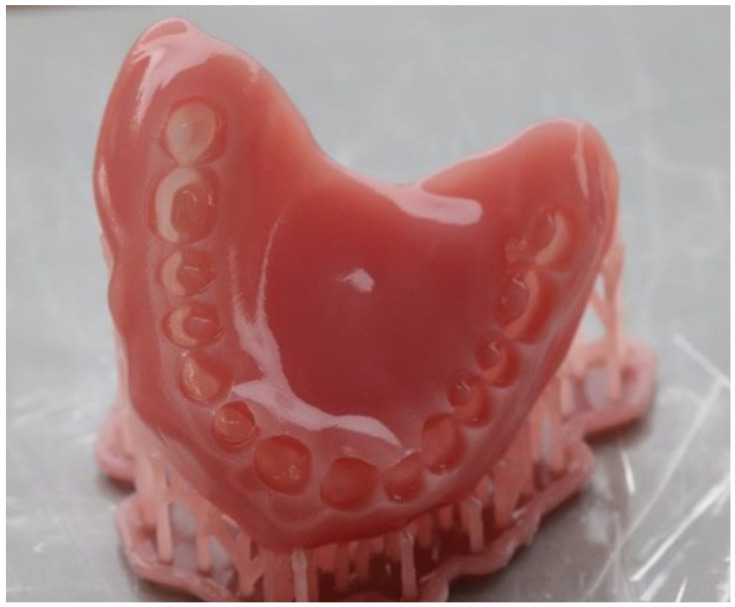
Three-dimensional-printed denture base for an upper complete removable denture (origin of the Figure: author’s own clinical case, no copyright issue), (NextDent, 3D Systems, Soesterberg, The Netherlands).

The process of printing through sintering or laser fusion (SLS) is quicker compared to alternative methods, albeit with higher costs. Within dental prosthetics, 3D printing serves multiple purposes: generating a model (from wax or plastic) that shall be transformed into the final prosthesis, or directly crafting definitive components from metal, resin, or ceramic materials (Figure 10) [87]. As of now, the extrusion technique, well-suited for thermoplastic polymers, finds primary use with PEEK [19]. Through an in vitro assessment of flexural strength (FS) values for six different prosthesis base resins, the following hierarchy was established: Machined resins (AvaDent and Polident) demonstrated superior results, trailed by a traditional heat-cured molded resin (Vertex) and a 3D-printed resin (NextDent, 3D Systems, Soesterberg, The Netherlands). In comparison, polyamide and another 3D-printed resin (Harz) exhibited notably lower values of flexural strength in contrast to standard resins [88].

### 4.3. Manufacturing of Artificial Teeth for Removable Dentures

**Figure 11:**
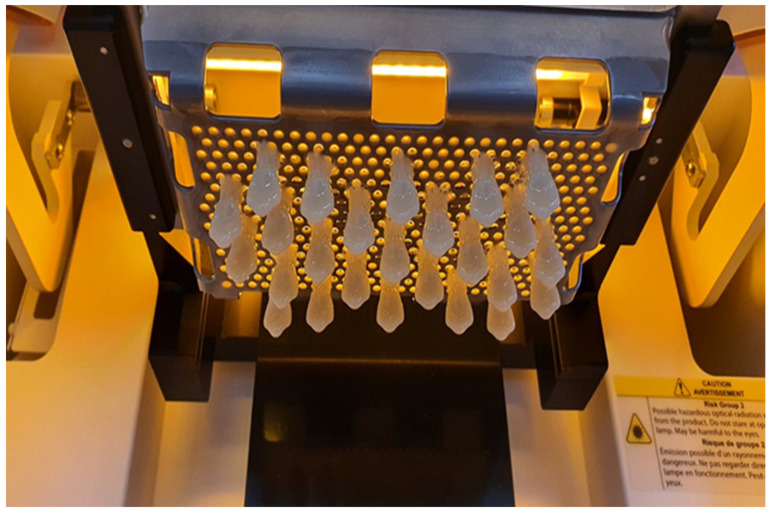
Three-dimensional printing of denture artificial teeth in the 3D printer NextDent 5100 (origin of the Figure: author’s own clinical case, no copyright issue), (NextDent, 3D Systems, Sosterberg, The Netherlands).

The STL file format is a recognized standard for conveying three-dimensional object geometry through triangular representations, commonly used for transmitting information to 3D printers [16]. NextDent Denture 3D + is a biocompatible dental resin utilized for various components of removable dentures, encompassing both the denture base and teeth [19]. The physical and mechanical attributes of this material closely resemble those of traditional acrylic resin applied in removable dentures [20]. The production of three-dimensional denture teeth involves a methacrylate-based photopolymerized resin, which is processed through 3D printing techniques [21]. The denture teeth and denture base are fabricated separately via 3D printing, followed by joining the printed teeth to the printed denture base using a light-cured bonding agent and undergoing a final additional polymerization step (Figure 11) [22].

### 4.4. Manufacturing of Occlusal Splints

**Figure 12:**
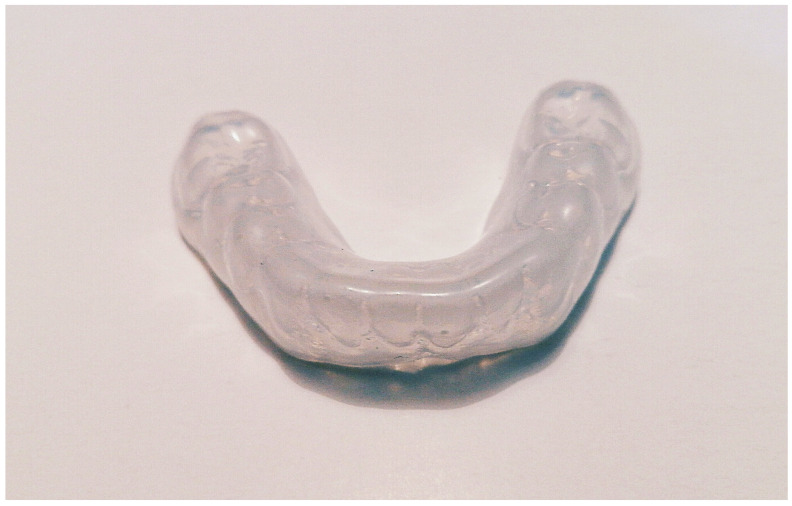
Three-dimensional–printed occlusal splint for the upper jaw (origin of the Figure: author’s own clinical case, no copyright issue), (NextDent Ortho Flex, 3D Systems, Soesterberg, The Netherlands).

In addition to the traditional scatter-and-press approach and subtractive milling technique, another avenue for crafting precisely fitting occlusal splints is through 3D printing (Figure 12) [109]. However, the accuracy of the overall production, as well as the material quality, long-term stability, and biocompatibility, play pivotal roles. Despite this, there is currently no substantial clinical experience with occlusal splints created through additive manufacturing. It is crucial to delve into the leaching behavior of additively manufactured occlusal splints both in laboratory settings and within the oral environment [64]. To determine the most effective long-term results, it would be valuable to compare this method with existing procedures [110].

The standards are notably high for uniformity and biocompatibility, standards often achieved by high-performance polymers produced through subtractive CAD/CAM methods, such as milled splints [34]. Elements like the arrangement and alignment of objects and their impact on precision, stability, and durability must also be thoroughly studied. The orientation of the objects on the build platform, and therefore the layering direction, appears to be particularly significant in this context. Initial research indicates that 3D-printed occlusal splints exhibit accuracy levels similar to CAD/CAM-milled splints, yet they demonstrate increased material wear and less favorable material properties [65,67].

## 5. Drawbacks of the Applications of Additive Manufacturing in Prosthetic Dentistry

Emerging technologies, particularly those related to component detachment, can effectively address the speed issue and result in exceptionally rapid construction speeds. However, constraints also exist in terms of the range of materials compatible with 3D printing [6]. Within the realm of polymers, dental technology predominantly relies on printers using photopolymer-based materials, especially those associated with VAT polymerization techniques (SLA, DLP, DUP). This narrows down the spectrum of resins that can be employed, creating notable drawbacks when compared to conventional manufacturing processes like CNC technologies and analog methods [10].

A potential solution to this predicament is the “drop-on-demand” technology, wherein medical-grade thermoplastics are melted from granulates and applied in a molten state, drop by drop, onto the build platform. However, the resulting surface quality achievable with this technique significantly diverges from the outcomes seen in filament printers [55]. Furthermore, there is a dearth of data concerning the behavior of 3D-printed devices or restorations within the oral environment. Limited information exists regarding plaque formation, material elution patterns, and the general biocompatibility of 3D-printed polymer materials [64,77]. Consequently, more extensive, and specific data on these aspects are urgently required.

Acrylic resins are the outcome of a polymerization process, wherein monomers undergo a heat, light, or chemically activated addition reaction to form stable polymers [111,112]. Despite the process, complete conversion from monomers to polymers is unattainable, leading to the presence of residual monomers and potentially harmful chemical by-products, such as methyl methacrylate, formaldehyde, methacrylic acid, benzoic acid, or dibutyl phthalate within the material [113,114,115,116]. These substances leach into the surrounding saliva through diffusion and subsequently interact with the host mucosa, potentially causing cytotoxic effects [117,118].

The degree of residual monomers is influenced by the polymerization method employed in the resin material’s manufacturing process. Dental materials produced by milling highly polymerized resin blanks exhibit residual monomer levels comparable to conventional materials [119]. In contrast, 3D-printed dental devices, cured through a light-based stepwise process, have been found to contain elevated levels of residual monomers [120]. Consequently, it seems plausible that 3D-printed oral splint materials may exert greater cytotoxicity than milled ones; however, surprisingly, scientific evidence supporting this notion is limited [121].

In essence, a distinction can be drawn between 3D printers designed for hobbyist use and those intended for professional applications. Practical experience has revealed that budget-friendly printers for hobbyists often yield unsatisfactory printing outcomes, particularly evident in the layering effect in FFF (fused-filament fabrication) printers due to filament fibers [29]. Consequently, the devices tailored for dental purposes tend to be more expensive yet produce satisfactory results. Nonetheless, even professional-grade printers exhibit some degree of layering effect in the Z-direction. This effect is largely influenced by the thickness of each individual layer. Thinner layers result in a reduced layering effect but also lead to extended processing times. Additionally, there are limitations concerning the maximum attainable print speed and the dimensions of the print area [101].

## 6. Conclusions

Additive processes offer a notable advantage by allowing the customization of an object’s properties during the construction phase. This influence extends to both mechanical and aesthetic attributes. In contrast, subtractive processes determine these qualities based on the characteristics of the milling blank used in manufacturing. Consequently, 3D printing empowers users with a wide array of choices even during the initial design stages. On the other hand, the precision and efficiency of subtractive machining are exceptionally high, which makes a combination of both manufacturing techniques a logical consideration. 3D-printing technologies have already been embraced in dental laboratories and practices for producing removable partial dentures (RPDs) using CoCr. Furthermore, an increasing number of publications are emerging on the additive manufacturing of complete dentures. The initial outcomes related to mechanical strength, fit, and surface quality are encouraging. Given that denture bases have extensive contact with the oral mucosa, meticulous evaluation of biocompatibility is necessary. Specifically, elution behavior and cytotoxicity must be thoroughly examined before reaching a conclusive assessment.

## Figures and Tables

**Table 1:** Three-dimensional-printed polymers, systemized by processing method and characteristics.

Type of Polymer	Processing Method	Characteristics
Methacrylic Acid (Formlabs: Dental SG)	Stereolithography	Elastic Modulus: 1670 MPa; Orientations of 0° to 90°.
Polylactic acid (PLA)	Fused deposition modeling	Tensile Strength: 28–56 Mpa; Elastic Modulus: 2000 Mpa; Orientations of 0° to 90°
Polyether ether ketone (PEEK)	Fused deposition modeling	Tensile Strength: 58–85 Mpa Elastic Modulus: 3000–4100 Mpa;
Acrylonitrile butadiene styrene (ABS)	Fused deposition modeling	Tensile Strength: 27–31 Mpa; Layer height: 0.05–0.14 mm; Processed at 210–240 °C. data

## Data Availability

Data are contained within the article.

## Abbreviations

ABS	acrylonitrile butadiene styrene
AM	additive manufacturing
CAD/CAM	computer-aided design/computer-aided manufacturing
CD	complete dentures
ISO	International Organization of Standardization
PC	polycarbonate
PCL	poly(e-caprolactone)
PDMS	polydimethylsiloxane
PE	polyethylene
PEEK	polyetheretherketone
PEG	polyethylene glycol
PLA	polylactic acid
PMMA	polymethyl methacrylate
PP	polypropylene
PU	polyurethane
STL	stereolithography, standard triangle language, standard tessellation language
3D	three-dimensional
